# Tissue-specific metabolomics and pigment profiling of Black Change and red Chal tomatoes

**DOI:** 10.1016/j.fochx.2026.103928

**Published:** 2026-04-27

**Authors:** Yu Miao, Mi-Bo Kim, Suhyeon Baek, Sanggil Lee, Lei Cao

**Affiliations:** a Department of Smart Green Technology Engineering, Pukyong National University, Busan, Republic of Korea; b Department of Food Science and Nutrition, College of Fisheries Science, Pukyong National University, Busan, Republic of Korea; c Ocean and Fisheries Development International Cooperation Institute, Pukyong National University, Busan, Republic of Korea

**Keywords:** Black Change tomato, Tissue-specific metabolomics, Flavonoid and phenolic profiling, Peel pigmentation, Chlorophyll retention

## Abstract

This study compared two Korean tomato cultivars, the black-pigmented Black Change tomato (BCT) and the red Chal tomato (RT), using tissue-resolved metabolomics and pigment analysis to clarify determinants of peel color and antioxidant potential. Untargeted UPLC–ESI–QTOF–MS profiling was integrated with external-standard quantification of naringenin chalcone and lycopene. BCT peel exhibited lower total phenolic/flavonoid contents and lower antioxidant capacity than RT peel, whereas cultivar differences in flesh and seeds were minor. RT peel contained more naringenin chalcone (6.68 vs 0.56 mg g^−1^ in BCT peel) and higher lycopene (426.88 vs 328.62 μg g^−1^), while rutin was more abundant in BCT. Pigment profiling showed strong chlorophyll retention in BCT, with chlorophyll *a* reaching 952.78 μg g^−1^ in peel, which likely masks carotenoids and produces the blackish appearance. Overall, the superior nutritional quality often attributed to BCT was not supported and BCT peel coloration is primarily associated with chlorophyll retention.

## Highlights

• Tissue-resolved metabolomics showed BCT–RT differences mainly in the peel.
• BCT peel showed lower antioxidant activity than red tomato (RT) peel.
• BCT dark color reflects retained chlorophyll, not superior antioxidant traits.
• RT peel was rich in naringenin chalcone; BCT peel accumulated rutin.
• Lycopene was higher in BCT flesh and seeds than RT, but higher in RT peel than BCT.

## Graphical abstract

**Figure:**
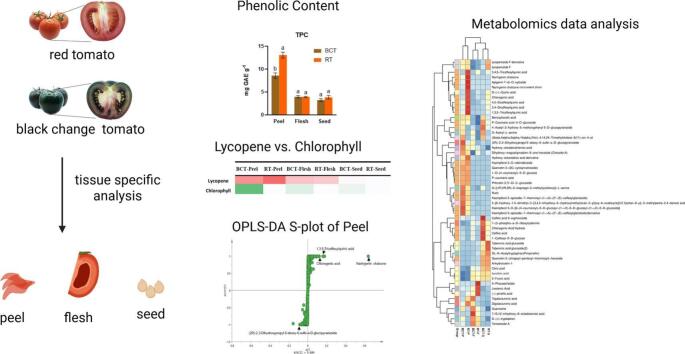

## 1 Introduction

Tomato (*Solanum lycopersicum* L.) is one of the most commonly consumed horticultural crops worldwide and serves as a major source of essential micronutrients and bioactive compounds in the human diet (Li et al., 2021). Extensive domestication and selective breeding have led to a substantial diversity of tomato cultivars with notable variations in color, shape, and taste. Among these traits, fruit color is a key attribute influencing both consumer acceptance and market value (Joung et al., 2025; Kurina et al., 2021). Tomato fruit color has attracted considerable interest also because it is closely associated with phytochemical variation among cultivars. In addition to conventional red tomatoes, yellow, orange, and black cultivars have been reported to exhibit distinct profiles of carotenoids, flavonoids, and other bioactive metabolites, resulting in substantial variation in nutritional and functional quality (Joung et al., 2025; Kurina et al., 2021). Although dark-colored tomatoes are often grouped together visually, their phytochemical composition and nutritional characteristics can differ markedly among cultivars (Blando et al., 2019; Joung et al., 2025).

Red Chal tomatoes (RT) are the most common tomato type in Korea, primarily characterized by their high lycopene content, a potent antioxidant carotenoid responsible for the red pigmentation. In comparison, black-hued cultivars such as the “Black Change tomato” (BCT) have been marketed in Korea as premium varieties with purportedly superior nutritional value, attributed to elevated levels of flavonoids such as rutin and naringenin chalcone. However, comparative evidence on the tissue-specific distribution of phytochemicals and pigments in BCT relative to conventional red tomatoes remains limited. Therefore, the present study aimed to systematically evaluate the nutritional composition and phytochemical profiles of BCT in comparison with conventional RT.

Previous studies have shown that tomato fruit exhibits marked spatial heterogeneity in metabolite composition, with peel and seed tissues contributing disproportionately to antioxidant pools relative to pulp; for instance, flavonoids and phenolic acids tend to accumulate in the peel, whereas organic acids and lipophilic constituents are more concentrated in the flesh and seeds (Bianchi et al., 2023; Moco et al., 2007). Therefore, this study employed a tissue-specific metabolomic approach by independently analyzing the peel, flesh, and seeds of RT and BCT. Utilizing ultra-performance liquid chromatography coupled with electrospray ionization quadrupole time-of-flight tandem mass spectrometry (UPLC-ESI-Q-TOF-MS) (Zhong et al., 2022), this study conducted an untargeted metabolomic analysis of phenolic and flavonoid constituents extracted with 70% methanol across distinct anatomical regions. This approach enabled a more precise interpretation of the unique color characteristics of the BCT and its association with pigment composition and antioxidant potential. Furthermore, this tissue-resolved metabolomic strategy offers novel insights into the metabolic heterogeneity of tomato fruit, enhancing the comprehensive evaluation of tomato nutritional properties.

## 2 Materials and methods

### 2.1 Reagents

LC/MS grade acetonitrile was used for UPLC-ESI-Q-TOF-MS. Ethyl acetate, and formic acid, and hydrochloric acid were purchased from Sigma-Aldrich (St. Louis, MO, USA). Methanol, ethanol, n-hexane, and acetic acid were obtained from Daejung (Seoul, Korea) Reagents used for antioxidant activity assays, including Folin-Ciocalteu's phenol reagent, 2,2-diphenyl-1-picrylhydrazyl (DPPH), phloroglucinol, FeCl₃, FeSO₄·7H_2_O, ascorbic acid, 2,2′-azino-bis (3-ethylbenzothiazoline-6-sulfonic acid) diammonium salt (ABTS), and 2,2′-azobis(2-amidinopropane) dihydrochloride (AAPH), were all obtained from Sigma-Aldrich.

### 2.2 Colorimetric measurement

BCT and RT were commercially purchased from an online retailer (Coupang, Korea). Three RT and three BCT fruits were randomly selected for colorimetric analysis. A colorimeter (TES Electrical Electronic Co., Ltd., Taipei, Taiwan) was used to measure the L^⁎^ (brightness), a^⁎^ (red/green), and b^⁎^ (yellow/blue) values from four sides and the bottom of each tomato (Arias et al., 2000). The device was calibrated against a standard white tile provided by the manufacturer. Overall color difference (ΔE) was calculated as follows.

(1) $$∆E=\sqrt{{\left(∆a\right)}^{2}+{\left(∆b\right)}^{2}+{\left(∆L\right)}^{2}}$$

### 2.3 Total antioxidant activity, Total phenolic and flavonoid content

Fresh tomatoes were rinsed with water to remove surface contaminants. Peel, flesh, and seeds were separated and homogenized using a blender. Homogenates were immediately frozen and freeze-dried. Freeze-dried tissues were stored at −20 °C until analysis.

Freeze-dried peel, flesh, and seed samples (200 mg) were extracted with 40 mL of 70% aqueous methanol using ultrasonication for 30 min. Extraction was repeated twice, and supernatants were combined. The pooled extracts were centrifuged at 4000 rpm for 5 min, filtered through a 0.45 μm PVDF syringe filter, and stored at −20 °C until analysis.

Total antioxidant capacity (TAC) was evaluated using DPPH, ABTS, and ferric reducing antioxidant power (FRAP) assays as previously described (Baek et al., 2025). Total phenolic content (TPC) was determined using a modified Folin-Ciocalteau's method (Koivikko et al., 2005), and total flavonoid content (TFC) was quantified by following the method of Suleria et al. (2020).

### 2.4 Anthocyanin detection

Anthocyanin analysis was conducted using the 70% methanol extracts of peel, flesh, and seed prepared as described in Section 2.3. Extracts were analyzed by HPLC on a C18 reversed-phase column using solvent A (deionized water containing 0.1% [*v*/v] formic acid) and solvent B (acetonitrile containing 0.1% [v/v] formic acid). The gradient elution program for solvent B was: 0–30 min, 5–30%; 30–50 min, 30–100%; 50–55 min, 100%; and 55–60 min, 100–5% for re-equilibration. Eluted compounds were monitored at 530 nm using a UV/PDA detector to assess anthocyanin-related signals.

### 2.5 UPLC–ESI–QTOF–MS/MS metabolite profiling

Metabolite profiling was performed using the 70% methanol extracts of peel, flesh, and seed prepared as described in Section 2.3. Metabolite analysis was performed using an UPLC system coupled to an ESI-enabled quadrupole time-of-flight mass spectrometer (SYNAPT XS, Waters, Milford, MA, USA). A pooled quality control (QC) sample was prepared by mixing equal aliquots of all tissue extracts and was injected periodically to monitor instrument stability. Separation was achieved with a Waters BEH C18 column (1.7 μm, 2.1 × 100 mm, Waters Corporation, Milford, MA, USA). Mobile phases were solvent A (water with 0.1% formic acid) and solvent B (acetonitrile with 0.1% formic acid). The gradient was: 0 min, 100:0 (A:B, *v*/v); 14 min, 0:100; 15 min, 100:0. Injection volume was 1 μL and flow rate was 0.3 mL/min. Column and sample temperatures were set at 40 °C and 15 °C, respectively.

Ionization was operated in negative mode. The capillary voltage was 2 kV and sampling cone voltage was 40 V (Pesaresi, Mizzotti, Colombo, & Masiero, 2014). Source and desolvation temperatures were 100 °C and 250 °C, respectively. Cone gas and desolvation gas flow rates were 50 and 600 L/h, respectively, with a nebulizer pressure of 6.5 bar. Full-scan spectra were acquired over the *m*/*z* range. Metabolites were tentatively identified using Progenesis QI (version 2.4, Nonlinear Dynamics, Waters Corporation) with chemical database ChemSpider.

### 2.6 Quantification of Naringenin Chalcone in tomato Peel

Naringenin chalcone was quantified from the 70% methanol peel extract prepared as described in Section 2.3. It was quantified using an ACQUITY UPLC system (Waters, Milford, MA, USA) coupled to a Xevo TQ mass spectrometer (Waters) and an Agilent Eclipse Plus C18 column (1.8 μm, 2.1 × 100 mm). Mobile phases were solvent A (water with 0.1% formic acid) and solvent B (acetonitrile with 0.1% formic acid). Flow rate was 0.30 mL/min, column temperature was 30 °C, and injection volume was 1 μL. The gradient was: 0–1 min, 90:10 (A:B); 7 min, 40:60; 8.5–9 min, 0:100; 10 min, 90:10; 11 min, re-equilibration at 90:10.

Mass detection was conducted using electrospray ionization (ESI) in negative ion mode ([M–H]^−^). Capillary voltage was 3.0 kV, source temperature 150 °C, and desolvation temperature 350 °C. Desolvation and cone gas flow rates were 500 and 60 L/h, respectively. Data were acquired in multiple reaction monitoring (MRM) mode using MassLynx (version 4.1, Waters). The transition *m*/*z* 271.1 → 119.1 was monitored with cone voltage 25 V and collision energy 25 eV. Quantification was performed using an external calibration curve (4, 6, 8, and 10 mg L^−1^) prepared from purified naringenin chalcone standard.

### 2.7 Quantification of lycopene

Lycopene extraction was performed in the dark to minimize degradation. Freeze-dried peel, flesh, and seed samples (40 mg each) were extracted with 10 mL of hexane:acetone:ethanol (50:25:25, *v*/v/v) by ultrasonication for 30 min. Distilled water (1.5 mL) was added to induce phase separation, and the upper organic phase (∼3 mL) was collected. Solvents were evaporated under reduced pressure at 30 °C. The residue was re-dissolved in 1 mL ethyl acetate and filtered through a 0.22 μm membrane filter prior to analysis.

The filtered samples were analyzed using Agilent Technologies 1200 Series HPLC-DAD system (Agilent Technologies, Santa Clara, CA, USA) and a C18 column (250 × 4.6 mm I.D., 5 μm, 12 nm; YMC Co., Ltd., Kyoto, Japan). Mobile phases were phase A (methanol) and phase B (ethyl acetate) (H.-K. Kim et al., 2015). The gradient was: 0 min, 20:80 (A:B); 4 min, 0:100; 6 min, 0:100; 7 min, 20:80. Injection volume was 20 μL. Quantification was based on an external standard calibration curve (0.2, 0.5, 1, 2, 5, and 10 mg L^−1^).

### 2.8 Calculation of chlorophyll content

Chlorophyll was extracted from freeze-dried tissue samples (200 mg) with 25 mL of 95% ethanol under dark conditions for 30 min. Extracts were centrifuged at 4000 rpm for 500 s, and absorbance of the supernatant was measured at 665 and 649 nm using 95% ethanol as the blank. Chlorophyll *a* and *b* concentrations were calculated using published equations (Qin & Wang, 2021):

(2a) $$\mathrm{chlorophyll}\;a\;\mathrm{concentration}\;\left(\mathrm{\mu g}\;g^{-1}\right)=\frac{13.75\times A_{665}-6.88\times A_{649}}{W}\times V\times 1000$$

(2b) $$\mathrm{chlorophyll}\;b\;\mathrm{concent}r\mathrm{ation}\;\left(\mathrm{\mu g}\;g^{-1}\right)=\frac{24.96\times A_{649}-7.32\times A_{665}}{W}\times V\times 1000$$

where A₆₆₅ and A₆₄₉ were the absorbance values at the respective wavelengths, V was the extract volume (mL), and W was the sample weight (mg).

### 2.9 Statistical analysis

All univariate experiments were conducted in triplicate, and results are expressed as mean ± standard deviation. Statistical significance was determined using one-way analysis of variance (ANOVA) followed by Tukey's post hoc test, performed with GraphPad Prism software (version 10.4.1, GraphPad Software Inc., La Jolla, CA, USA). Differences were considered statistically significant at *p* < 0.05.

For metabolomics, raw intensity values were preprocessed using variance stabilizing normalization to reduce technical variation. Homogeneity of variance was assessed using Levene's test (*p* > 0.05). Multivariate analyses were performed using SIMCA (version 14.1, Umetrics, Kinnelon, NJ, USA). PCA was used to examine global variation and outliers, and OPLS-DA was used to maximize group separation. Model validity was evaluated using cross-validation parameters (R^2^, Q^2^) and permutation tests. Metabolites were considered significant if they met VIP > 2.0 and FDR-adjusted p < 0.05 (one-way ANOVA with Benjamini–Hochberg correction). Hierarchical clustering analysis and heatmap visualization were conducted in R (version 4.4.1).

## 3 Results

### 3.1 Measurement of the colorimetry parameters

**Fig. 1:**
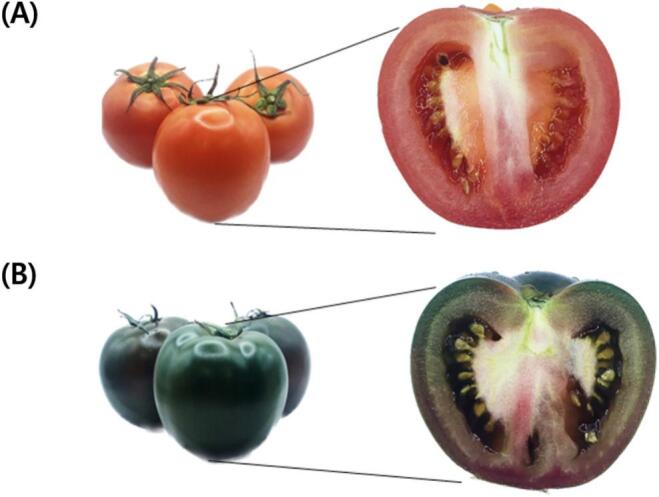
Representative front and cross-sectional images. (A) External view (front) and half-section of RT and (B) BCT.

Colorimetry confirmed clear surface color differences between RT and BCT (Fig. 1; Table 1). RT exhibited significantly higher values in all three parameters, L^⁎^ (brightness), a^⁎^ (red/green), and b^⁎^ (yellow/blue), indicating a brighter and more red/yellow appearance, whereas BCT showed a negative a* value, consistent with a greener/darker hue. The resulting ΔE value between RT and BCT was 39.89, indicating a pronounced overall color difference.

**Table 1:** Color measurement values of two tomato varieties.

Category	L*	a*	b*
RT	39.41 ± 2.18 ^a^	30.37 ± 0.75 ^a^	29.17 ± 1.11 ^a^
BCT	33.51 ± 0.08 ^b^	−5.38 ± 2.29 ^b^	22.04 ± 2.20 ^b^

RT, red Chal tomato; BCT, Black Change tomato. Values within the same column that do not share a common superscript letter are significantly different (*p* < 0.05).

### 3.2 TAC, TPC, TFC and anthocyanins

**Fig. 2:**
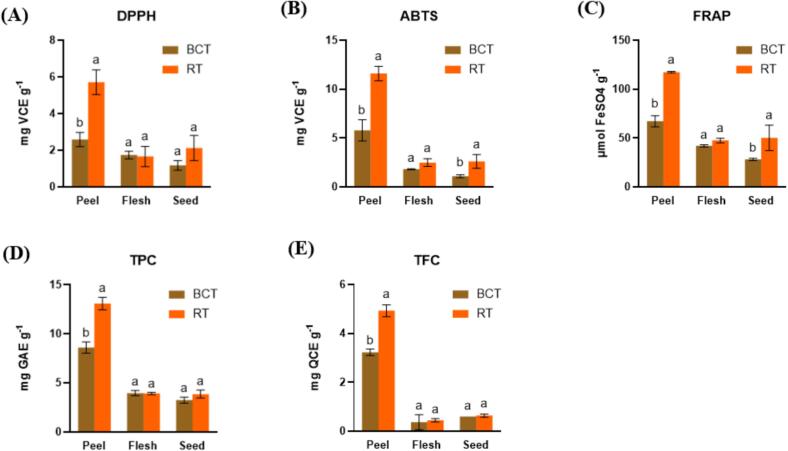
TPC, TFC, and TAC of 70% methanol extracts from different parts of two tomatoes. (A) DPPH, (B) ABTS, (C) FRAP, (D) TPC, and (E) TFC.

BCT has been reported as having superior phenolic composition, including higher levels of rutin, naringenin chalcone, and quercetin, compared with comparator tomato types (G. Kim, 2018). To evaluate this, TAC (DPPH, ABTS, FRAP), TPC, and TFC were measured in peel, flesh, and seed extracts (Fig. 2). Across both cultivars, peel consistently showed the highest TAC/TPC/TFC. RT peel exhibited significantly stronger antioxidant capacity than BCT peel in all three TAC assays, together with higher TPC and TFC. In contrast, no significant differences were detected between the two tomato varieties in the flesh fraction for TAC, TPC, or TFC. However, in seeds, RT showed higher ABTS and FRAP than BCT, while DPPH, TPC, and TFC did not differ. Notably, anthocyanins tested negative in both BCT and RT using the pH-shift method, and no clear peaks were observed at 530 nm in the HPLC chromatograms; therefore, no quantitative data are presented.

It should be noted that these TAC assays reflect in vitro chemical antioxidant capacity under defined assay conditions and may not directly translate to antioxidant effects in vivo, which depend on factors such as bioaccessibility, absorption, metabolism, and tissue distribution.

### 3.3 Metabolomics analysis of different parts of BCT and RT

#### 3.3.1 Identification of differentially accumulated metabolites (DAMs) in tomato

Untargeted profiling indicated tissue-dependent metabolic variation, with the strongest varietal separation in peel (Figs. S1–S2). Metabolite annotation revealed a broad spectrum of compounds, including amino acids and their derivatives, organic acids, nucleotides, fatty acids, glycosides, saponins, terpenoids, polysaccharide derivatives, and diverse phenolics such as flavonoids (Table S1). Many phenolic compounds, such as kaempferol and quercetin derivatives, were found in glycosylated forms. For example, kaempferol 3-O-robinobioside and kaempferol 3-O-[6-(4-coumaroyl)-β-D-glucosyl-(1 → 2)-β-D-glucosyl-(1 → 2)-β-D-glucoside] are kaempferol-derived compounds. In addition to flavonoids, several organic acids were detected in both RT and BCT, including citric acid, aconitic acid, citraconic acid, and isocitric acid. Many of these organic acids are central intermediates of the tricarboxylic acid (TCA) cycle, a key pathway in primary metabolism. In addition, several fatty acids, such as linolenic acid and (−)-pinellic acid, and steroidal glycoalkaloids, such as lycoperoside F and tomatoside A were also identified based on their characteristic fragmentation patterns (Otify et al., 2023; Yamanaka et al., 2008).

**Fig. 3:**
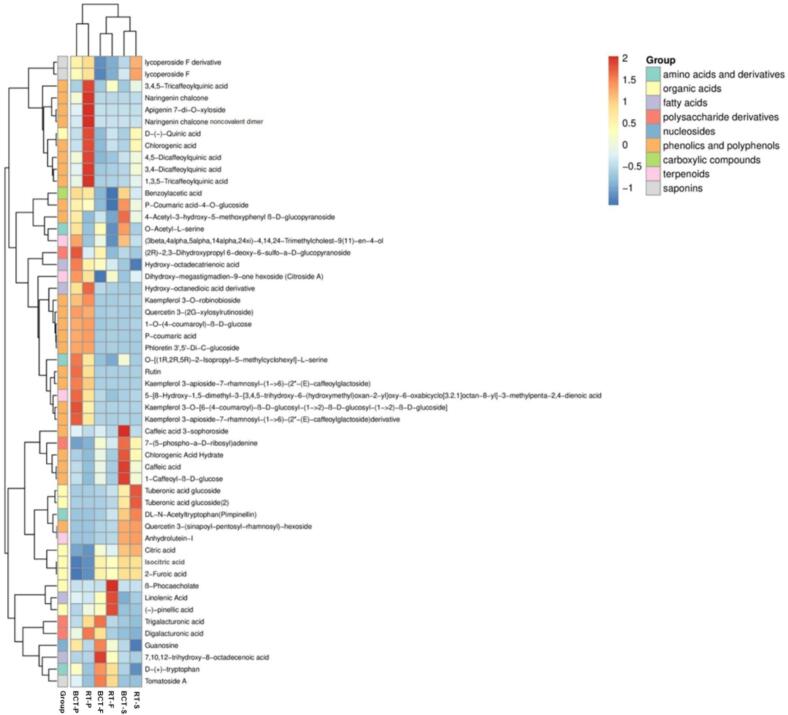
Heatmap of differential metabolites identified in peel, flesh, and seed of BCT and RT.

To identify metabolites that contributed most significantly to group separation, OPLS-DA was conducted separately for peel, flesh, and seed samples. The resulting models showed high goodness-of-fit and predictive performance (peel: R^2^X(cum) = 0.7336, R^2^Y(cum) = 0.9999, Q^2^ = 0.9849; flesh: R^2^X(cum) = 0.7306, R^2^Y(cum) = 0.9979, Q^2^ = 0.8023; seed: R^2^X(cum) = 0.6097, R^2^Y(cum) = 0.9928, Q^2^ = 0.7101). In total, 53 DAMs were identified across the three tissue types (Table 2), including 32 in peels, 31 in flesh, and 29 in seeds. Several metabolites were shared among two or more tissues, indicating partial overlap between groups. To visualize DAM patterns across tissues and tomato types, hierarchical clustering analysis and heatmap analyses were performed (Fig. 3), revealing clear differences in metabolite profiles. Clear distinctions were observed across tissue types. Notably, phenolic compounds were most abundant in the peel, whereas lipid-related metabolites were highly enriched in the seeds. Peel samples displayed the most pronounced varietal differences, particularly in phenolic compound, with BCT peel showing higher relative intensities for multiple flavonoid glycosides (e.g., kaempferol- and quercetin-based derivatives) compared to RT peel. Organic acids, including citric acid and isocitric acid, were more abundant in flesh and seed samples, with some variety-dependent differences. Fatty acids such as linolenic acid and (−)-pinellic acid were generally higher in seeds, particularly RT seed.

**Table 2:** Tentatively identified DAM in peel, flesh, and seed of two tomato types.

	Proposed compound	RT (min)	[M − H] ^–^(m/z)	MS/MS (m/z)	Class	DAM among
1	O-Acetyl-l-serine	0.82	146.0459	146,132,115	Amino acids and derivatives	Peel, flesh, and seed
2	Citric acid	0.87	191.0201	191,173	Organic acids	Peel, flesh, and seed
3	Linolenic Acid	0.90	277.0316	277,191	Fatty acids	Flesh
4	Trigalacturonic acid	0.92	545.1011	545,317	Polysaccharide derivatives	Peel and flesh
5	Digalacturonic acid	0.92	369.0676	369,278,223	Polysaccharide derivatives	Peel and flesh
6	(2*R*)-2,3-Dihydroxypropyl 6-deoxy-6-sulfo-α-D-glucopyranoside	0.95	317.055	317,225	Polysaccharide derivatives	Peel and flesh
7	7-(5-phospho-α-D-ribosyl) adenine	1.50	346.0561	346,134	Polysaccharide derivatives	Flesh and seed
8	Isocitric acid	1.81	191.0201	110,173,191	Organic acids	Peel and flesh
9	2-Furoic acid	1.81	111.0082	111,87	Organic acids	Flesh
10	Guanosine	3.39	282.0846	282,150,108	Nucleosides	Flesh and seed
11	Caffeic acid	4.47	179.0350	179,135	Phenolics and polyphenols	Flesh and seed
12	1-Caffeoyl-β-d-glucose	4.47	341.0884	135,179,341	Phenolics and polyphenols	Peel, flesh, and seed
13	Benzoylacetic acid	4.53	163.0401	163,119	Organic acids	Flesh and seed
14	Chlorogenic Acid Hydrate	4.53	371.0986	163,119	Phenolics and polyphenols	Flesh and seed
15	P-Coumaric acid-4-O-glucoside	4.53	325.0931	163,119	Phenolics and polyphenols	Flesh and seed
16	D-(+)-tryptophan	4.71	203.0827	203,142,116	Amino acids and derivatives	Peel, flesh, and seed
17	5-[8-Hydroxy-1,5-dimethyl-3-[3,4,5-trihydroxy-6-(hydroxymethyl) oxan-2-yl] oxy-6-oxabicyclo [3.2.1] octan-8-yl]-3-methylpenta-2,4-dienoic acid	4.95	443.1926	443,387	Polysaccharide derivatives	Peel and flesh
18	4-Acetyl-3-hydroxy-5-methoxyphenyl β-D-glucopyranoside	4.95	327.103	181,113	Phenolics and polyphenols	Flesh and seed
19	D-(−)-Quinic acid	5.15	191.0563	111,127,173	Organic acids	Peel and seed
20	Chlorogenic acid	5.15	353.0880	191,353	Phenolics and polyphenols	Peel, flesh, and seed
21	p-Coumaric acid	5.34	163.0402	119,163	Phenolics and polyphenols	Flesh
22	1-O-(4-coumaroyl)-β-d-glucose	5.34	325.0932	119,163	Phenolics and polyphenols	Flesh
23	Tuberonic acid glucoside	5.54	387.1661	387,163,300	Saponins	Seed
24	Hydroxy-octanedioic acid derivative	5.61	351.1298	351,300,241	Fatty acids	Peel
25	Tuberonic acid glucoside (2)	5.84	387.1662	191,387	Saponins	Flesh and seed
26	Dihydroxy-megastigmadien-9-one hexoside (Citroside A)	5.96	385.1927	385,365	Terpenoids	Flesh
27	(3beta,4alpha,5alpha,14alpha,24xi)-4,14,24-Trimethylcholest-9(11)-en-4-ol	6.02	427.1826	389,381	Terpenoids	Peel, flesh, and seed
28	Quercetin 3-(2G-xylosylrutinoside)	6.34	741.1893	300,740	Phenolics and polyphenols	Peel
29	Rutin	6.74	609.1465	300,609	Phenolics and polyphenols	Peel
30	Phloretin 3′,5’-Di-C-glucoside	7.03	597.183	597,387,357	Phenolics and polyphenols	Peel
31	DL-N-Acetyltryptophan (Pimpinellin)	7.23	245.0935	245,203	Amino acids and derivatives	Flesh and seed
32	Lycoperoside F derivative	7.25	1291.5975	1268,1136	Saponins	Seed
33	Kaempferol 3-O-robinobioside	7.25	593.1517	179,285,515,593	Phenolics and polyphenols	Peel and seed
34	Lycoperoside F	7.25	1268.5942	1268,1136	Saponins	Seed
35	4,5-Dicaffeoylquinic acid	7.38	515.1205	179,191,353,515	Phenolics and polyphenols	Peel and seed
36	Quercetin 3-(sinapoyl-pentosyl-rhamnosyl)-hexoside	7.60	947.2484	947, 741, 271	Phenolics and polyphenols	Peel
37	Anhydrolutein-I	7.62	549.1987	549,387	Terpenoids	Seed
38	Kaempferol 3-O-[6-(4-coumaroyl)-β-D-glucosyl-(1- > 2)-β-D-glucosyl-(1- > 2)-β-D-glucoside]	7.80	917.2373	300,723,741,887,917	Phenolics and polyphenols	Peel
39	Kaempferol 3-apioside-7-rhamnosyl-(1- > 6) -(2″-(*E*)-caffeoylglactoside)	7.80	887.2261	300,723,741,887	Phenolics and polyphenols	Peel
40	3,4-Dicaffeoylquinic acid	7.86	515.12	135,179,353,515	Phenolics and polyphenols	Peel and seed
41	Caffeic acid 3-sophoroside	8.04	503.178	448,163	Phenolics and polyphenols	Seed
42	Kaempferol 3-apioside-7-rhamnosyl-(1- > 6) -(2″-(E)-caffeoylglactoside) derivative	8.49	921.2682	271,300,723,741, 877,000	Phenolics and polyphenols	Peel
43	3,4,5-Tricaffeoylquinic acid	8.58	677.2838	677,645,520,488	Phenolics and polyphenols	Peel, flesh, and seed
44	Tomatoside A	8.79	1081.5470	757,919	Terpenoids	Peel
45	1,3,5-Tricaffeoylquinic acid	9.26	677.1522	677,515	Phenolics and polyphenols	Peel and seed
46	Apigenin 7-di-O-xyloside	10.17	565.1122	565,271	Phenolics and polyphenols	Peel
47	Naringenin chalcone	10.17	271.0619	271,151,119	Phenolics and polyphenols	Peel, flesh, and seed
48	Naringenin chalcone noncovalent dimer	10.17	543.1302	543, 271,151,119	Phenolics and polyphenols	Peel
49	O-[(1*R*,2*R*,5*R*)-2-Isopropyl-5-methylcyclohexyl]-l-serine	10.85	242.1759	242,225,181	Amino acids and derivatives	Peel
50	(−)-pinellic acid	11.19	329.2335	329,229,171	Organic acids	Flesh
52	7,10,12-Trihydroxy-8-octadecenoic acid	12.52	329.2335	329,201	Fatty acid	Flesh
53	β-Phocaecholate	12.97	407.2802	407,279	Organic acids	Flesh
54	Hydroxy-octadecatrienoic acid	14.62	293.1766	293,249,193	Organic acids	Peel

#### 3.3.2 Multivariate analyses of peels of BCT and RT varieties

**Fig. 4:**
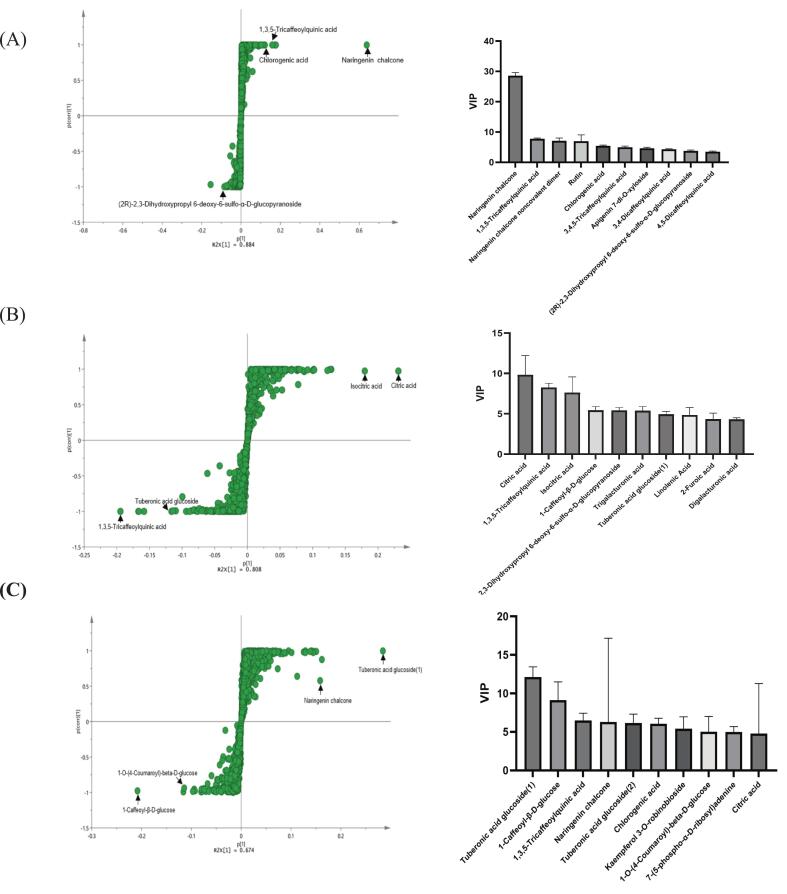
Multivariate analysis of major metabolites in peel, flesh, and seed of BCT and RT varieties. OPLS-DA S-plot indicating key metabolites contributing to group separation and the top 10 metabolites ranked by VIP score in (A) peel, (B) flesh, and (C) seed of BCT and RT.

Fig. 4A presented OPLS-DA-based multivariate analysis of metabolite profiles in the peels of RT and BCT. The S-plot identified the metabolites with the greatest discriminatory power. Notably, naringenin chalcone, 1,3,5-tricaffeoylquinic acid, and chlorogenic acid showed strong association with RT peel. In contrast, (2*R*)-2,3-dihydroxypropyl 6-deoxy-6-sulfo-α-D-glucopyranoside was more enriched in BCT peel and contributed oppositely to the group separation. The VIP score plot further emphasized these trends, ranking naringenin chalcone as the top metabolite (VIP score 28.55). This emphasized its dominant role in differentiating peel samples from different varieties. Another highly ranked flavonoid was rutin, which were more abundant in BCT, suggesting alternative flavonoid accumulation patterns between the two varieties.

#### 3.3.3 Multivariate analyses of flesh of BCT and RT varieties

Fig. 4B illustrated the OPLS-DA-based metabolomic comparison of the flesh tissues between RT and BCT. The S-plot identified key metabolites responsible for group separation. Among them, citric acid and isocitric acid showed strong association with RT, while 1,3,5-tricaffeoylquinic acid and tuberonic acid glucoside were more enriched in BCT. The VIP score plot ranked the top contributors to varietal discrimination in tomato flesh. Citric acid, isocitric acid, 1,3,5-tricaffeoylquinic acid, and 1-caffeoyl-β-d-glucose exhibit the highest VIP values. These compounds represent a mix of primary metabolites (e.g., citric acid and isocitric acid) and secondary phenolic derivatives, showing that both were important in distinguishing the two tomato types.

#### 3.3.4 Multivariate analyses of seed of BCT and RT varieties

The OPLS-DA S-plot identified key metabolites that separated RT and BCT seeds (Fig. 4C). Tuberonic acid glucoside was strongly associated with BCT seeds. In contrast, 1-caffeoyl-β-d-glucose and 1-O-(4-coumaroyl)-β-d-glucose were more linked to RT seeds. The VIP score plot confirmed tuberonic acid glucoside as the top-ranked metabolite. Other influential contributors included 1-caffeoyl-β-d-glucose and naringenin chalcone, which also played important roles in varietal separation.

#### 3.3.5 Phenolic acid and flavonoid synthesis pathway

**Fig. 5:**
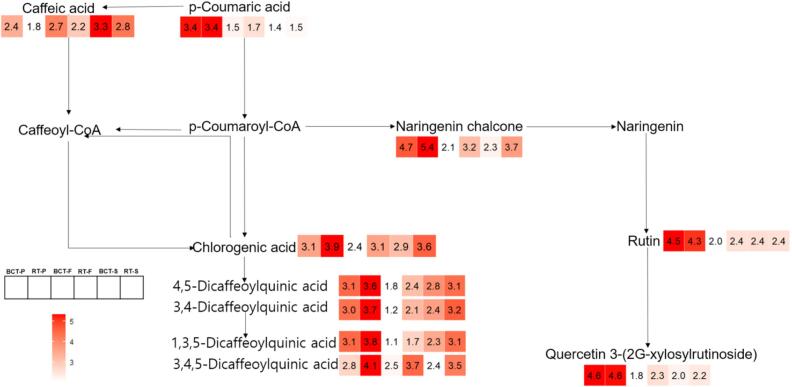
Metabolite mapping on the phenolic acid and flavonoid synthesis pathway and comparison between six different tomato cultivars, including peel (BCT-P and RT-P), flesh (BCT-F and RT-F), and seed (BCT-S and RT-S) of BCT and RT. Heatmaps were expressed by Log10 of means of the compound concentration.

Fig. 5 mapped the phenylpropanoid-flavonoid pathway of six tomato tissue samples. The heatmap displayed ion-peak intensities after log10 transformation, revealing distinct tissue- and variety-specific patterns of metabolite accumulation. Peels from both varieties were enriched in most flavonoids, including naringenin chalcone, rutin, and quercetin 3-(2G-xylosylrutinoside). Chlorogenic acid and several dicaffeoylquinic acids also showed their highest levels in peel, followed by seed and flesh. In contrast, caffeic acid exhibited the opposite trend, being most abundant in seed, moderate in flesh, and lowest in peel.

Varietal differences were also pronounced. Naringenin chalcone levels in BCT peel, flesh, and seed were only 20%, 8%, and 4% of those in RT, respectively. External standard quantification confirmed this difference: naringenin chalcone in BCT peel was 0.56 mg g^−1^, which was 8.38% of the RT peel concentration (6.68 mg g^−1^) (Fig. S3). Quercetin 3-(2G-xylosylrutinoside) and rutin were also lower in BCT flesh.

In the phenolic acid pathway, chlorogenic acid and multiple dicaffeoylquinic acid isomers (e.g., 4,5-, 3,4-, and 3,4,5-dicaffeoylquinic acids) were consistently lower in all three BCT tissues than in RT. By contrast, caffeic acid accumulated more in BCT.

### 3.4 Lycopene determination

Lycopene is the predominant carotenoid in ripe tomatoes, accounting for approximately 80–90% of the total carotenoid content, and it is the principal determinant of the fruit's red coloration (Ilahy et al., 2011). The peel of RT contained 426.88 ± 0.66 μg g^−1^ of lycopene, significantly higher than the 328.62 ± 5.28 μg g^−1^ detected in the BCT peel (Table 3). Conversely, the flesh and seeds of BCT exhibited slightly higher lycopene contents (196.52 ± 2.56 μg g^−1^ and 61.34 ± 0.52 μg g^−1^, respectively) compared to those of RT (182.74 ± 6.58 μg g^−1^ and 49.89 ± 1.16 μg g^−1^, respectively).

**Table 3:** Determination of lycopene and chlorophyll in different parts of BCT and RT.

	BCT-P	RT-P	BCT-F	RT-F	BCT-S	RT-S
Lycopene (μg g^−1^)	328.62 ± 5.28 ^b^	426.88 ± 0.66 ^a^	196.52 ± 2.56 ^c^	182.74 ± 6.58 ^d^	61.34 ± 0.52 ^e^	49.89 ± 1.16 ^f^
Chlorophyll *a* (μg g^−1^)	952.78 ± 1.33 ^a^	8.16 ± 1.01 ^e^	190.52 ± 0.50 ^b^	2.33 ± 1.01 ^f^	116.52 ± 0.86 ^c^	13.15 ± 0.87 ^d^
Chlorophyll *b* (μg g^−1^)	307.79 ± 2.12 ^a^	1.06 ± 0.53 ^d^	66.38 ± 1.80 ^b^	N/D	64.29 ± 3.12 ^b^	8.23 ± 1.60 ^c^

BCT-P, Black Change tomato peel; RT-P, red Chal tomato peel; BCT—F, Black Change tomato flesh; RT-F, red Chal tomato flesh; BCT—S, Black Change tomato seed; RT-S, red Chal tomato seed; N/D, not detected. Values within the same column that do not share a common superscript letter are significantly different (*p* < 0.05).

### 3.5 Determination of chlorophyll

Chlorophyll is typically abundant in immature tomatoes and gradually degrades during ripening, leading to the visual transition from green to red. In this study, fully ripened fruits showed markedly higher concentrations of both chlorophyll *a* and chlorophyll *b* in BCT compared with RT (Table 3). For chlorophyll *a*, BCT contained 952.78 μg g^−1^ in peel, 190.52 μg g^−1^ in flesh, and 116.52 μg g^−1^ in seeds, while RT tissues contained only trace amounts (≤ 15 μg g^−1^). A similar pattern was observed for chlorophyll *b*: BCT measured 307.79 μg g^−1^ in peel, 66.38 μg g^−1^ in flesh, and 64.29 μg g^−1^ in seeds, whereas RT showed minimal levels (≤ 8.23 μg g^−1^). These findings indicated that BCT retained substantial chlorophyll in all tissues at full ripeness, especially in peel, suggesting incomplete chlorophyll degradation in BCT.

## 4 Discussion

This study presents a detailed comparative analysis of RT and BCT, focusing on tissue-specific differences in colorimetric properties, antioxidant capacities, and metabolite profiles. While black tomato is often marketed as a premium variety with superior nutritional properties, our findings challenge this assumption by providing a comprehensive biochemical characterization across peel, flesh, and seed tissues.

Colorimetric analysis revealed distinct differences in surface coloration between the two cultivars. The L^⁎^ value, representing brightness, was lower in BCT, indicating a darker appearance, while the a^⁎^ value, which denotes the red-green axis, was positive in RT and negative in BCT, reflecting a greener hue. The b^⁎^ value, associated with the yellow-blue axis, was also higher in RT, suggesting more yellow pigmentation. Moreover, the ΔE value (39.89) indicates a pronounced color difference between BCT and RT (Minaker et al., 2021).

Typically, L^⁎^ decreases as tomatoes ripen, and values below 40 generally indicate maturity and high lycopene content (Arias et al., 2000). Consistent with this, both cultivars showed mature fruit profiles with substantial lycopene content across tissues. The overall lycopene content was higher in BCT than in RT. However, the tissue-specific distribution of lycopene varied: BCT contained higher lycopene levels in the flesh and seeds compared to RT counterparts, but exhibited significantly lower lycopene in the peel than RT. This pattern suggests that carotenoid biosynthesis or accumulation in the peel of BCT may be selectively suppressed, despite active production in internal tissues. A plausible explanation is the marked retention of chlorophyll observed in the BCT peel. Even at full ripeness, BCT peel retained nearly 1000 μg g^−1^ of chlorophyll *a* and around 300 μg g^−1^ of chlorophyll *b*, while RT tissues contained only trace amounts. Normally, chlorophyll degrades during ripening as chloroplasts differentiate into chromoplasts (Cheung et al., 1993). This degreening process depends on coordinated control of plastid remodeling and the chlorophyll catabolic pathway, such that delayed activation or partial impairment of key catabolic steps can result in chlorophyll retention. Consistent with this mechanism, loss of function of the Mg-dechelatase STAY-GREEN, which acts early in chlorophyll breakdown, has been reported to confer a chlorophyll retention phenotype in ripening tomato fruits (Zheng et al., 2025). The co-existence of high chlorophyll and moderate lycopene in BCT peel likely results in optical masking of red pigmentation, leading to the observed negative a^⁎^ value and the characteristic dark appearance.

In terms of antioxidant potential, tissue-specific assays (DPPH, ABTS, and FRAP) and measurements of total phenolic and flavonoid contents demonstrated that BCT peel consistently exhibited lower antioxidant activity than RT peel, while differences between cultivars were minor in flesh and seeds. This contrasts with black-hued cultivars such as Sun Black, which display nearly twofold higher TPC than red tomatoes due to anthocyanin accumulation (Blando et al., 2019). In our samples, however, anthocyanins were not detected in the peel or other tissues of either BCT or RT, indicating that anthocyanins are unlikely to contribute to the cultivar-dependent differences observed here.

Metabolomic profiling provided further insights into these tissue-specific differences. RT peel contained significantly higher levels of naringenin chalcone and chlorogenic acid, two key intermediates in flavonoid and phenolic biosynthesis in tomato. Because phenolic acids and flavonoids contribute substantially to DPPH and ABTS scavenging and FRAP responses, the coordinated enrichment of these metabolites in RT peel provides a mechanistic basis for its higher TAC together with elevated TPC and TFC. Naringenin chalcone tends to accumulate in tomato peel and can be converted into downstream flavonoids, including naringenin, kaempferol, quercetin, and their glycosides, via the coordinated action of enzymes such as chalcone isomerase, flavanone 3-hydroxylase, and flavonol synthase. These compound classes are closely associated with antioxidant function and pigmentation (D. S. Kim et al., 2014), and their enrichment in RT peel corresponded well with its higher TPC and TFC values. Flavonoids, in particular, are recognized for their antioxidant properties and yellow pigmentation, which contributes to coloration in certain yellow tomato cultivars (Slimestad et al., 2008; Spencer et al., 2005; Yan Zhang et al., 2017). In tomato fruit, flavonoid biosynthesis is primarily active in the peel, while remaining largely inactive in the flesh due to minimal expression of key biosynthetic genes (Schijlen et al., 2006). The high concentration of flavonoids in RT peel was also consistent with its elevated b^⁎^ value in colorimetric evaluations, reflecting a more intense yellow hue compared to the BCT peel.

Flavonoids and phenolic acids are two major subclasses of polyphenolic compounds in tomatoes, both synthesized from the common phenylpropanoid precursor p-coumaric acid (Marchiosi et al., 2020; Tudor-Radu et al., 2016). This metabolite serves as a crucial branching point for metabolic flux. In the phenolic acid pathway, p-coumaric acid is hydroxylated by p-coumarate 3′-hydroxylase (C3′H) to form caffeic acid, which is subsequently esterified to produce chlorogenic acid and related derivatives. Alternatively, p-coumaric acid can be activated to p-coumaroyl-CoA, which enters the flavonoid pathway to generate chalcones and other flavonoid derivatives (Islam et al., 2019).

Consistent with this branching, chlorogenic acid and its derivatives were substantially enriched in RT peel, suggesting that in this cultivar p-coumaroyl-CoA may be preferentially diverted toward the phenolic acid branch. Notably, these interpretations are inferred from relative abundance patterns rather than from direct measurements of enzyme activity, gene expression, or metabolic flux. In contrast, BCT peel exhibited higher levels of rutin (quercetin-3-O-rutinoside), a quercetin glycoside, indicating that downstream flavonoid glycosylation pathways were more active in this cultivar despite lower precursor accumulation. Since rutin is synthesized through stepwise glycosylation of quercetin by uridine diphosphate-dependent glycosyltransferases (Zou et al., 2023), its relative abundance in BCT highlights cultivar-specific differences in the balance between phenolic acid production and flavonoid glycosylation. Together, these results support the inference that differences in metabolite partitioning at the p-coumaroyl-CoA branching point are associated with the distinct flavonoid and phenolic profiles observed between RT and BCT.

The flesh and seed tissues, by comparison, showed less pronounced varietal divergence. In the flesh, organic acids such as citric acid and isocitric acid were the main contributors to differences between RT and BCT. Although BCT has been reported as having higher levels of certain flavonoids, our flesh extracts did not show elevated rutin, naringenin chalcone, or quercetin relative to RT. Overall, the flesh and seed contained fewer secondary metabolites, such as phenolics and flavonoids, than the peel. Instead, organic acids are among the most abundant and functionally significant metabolites in tomato fleshes, playing a critical role in determining organoleptic properties, particularly acidity and overall flavor perception (Cheng et al., 2020; Yilmaz, 2001). In particular, their involvement in energy-generating pathways such as the TCA cycle necessitates homeostatic control, resulting in limited inter-varietal variability (Youjun Zhang & Fernie, 2023).

Glycosylated metabolites, including tuberonic acid glucoside and 1-caffeoyl-β-d-glucose, were important discriminants in seeds. RT seeds showed higher levels of tuberonic acid glucoside, a glycosylated derivative of jasmonic acid that has been associated with developmental regulation, dormancy, and stress signaling (Wasternack & Song, 2017). In contrast, BCT seeds contained more 1-caffeoyl-β-d-glucose, a glycosylated form of caffeic acid that is often linked to improved metabolite stability and storage (Jones & Vogt, 2001). Such a pattern could reflect a metabolic preference in BCT toward stabilizing phenolic acids rather than producing jasmonate-derived signaling compounds (Kytidou et al., 2020; Le Roy et al., 2016). While the precise physiological implications remain to be elucidated, these metabolite profiles suggest potentially distinct strategies for secondary metabolite regulation and storage in seeds between the two cultivars.

Although these differences are significant at the tissue level, their nutritional implications for human consumption should be considered in context. The peel and seeds represent only a small fraction of total fruit weight (about 4% in a 130 g fresh tomato) (Del Valle et al., 2006), meaning that their contribution to dietary antioxidant intake is limited. Therefore, while RT and BCT differ in pigment composition and antioxidant distribution, their overall nutritional value in typical dietary portions may be more similar than the visual differences suggest. A limitation of this work is that tissues were pooled and analyzed in technical triplicate, rather than using independent biological replicates from multiple batches or manufactures; therefore, the findings should be interpreted as differences observed in the analyzed samples and not as definitive cultivar-level variation.

This work contributes to the United Nations' Sustainable Development Goals (SDGs) by providing evidence-based nutritional communication for tomatoes, supporting improved quality control and responsible agri-food innovation.

## 5 Conclusion

This study shows that BCT's black peel is largely associated with chlorophyll retention, rather than reflecting enhanced antioxidant value. Tissue-resolved analyses showed that cultivar differences were most pronounced in the peel, while RT exhibited higher phenolic levels and antioxidant capacity, consistent with its higher abundance of naringenin chalcone. In contrast, BCT was characterized by strong chlorophyll retention that likely masks carotenoid-derived redness. Together, these results caution against using tomato peel color alone as a nutritional proxy.

## CRediT authorship contribution statement

**Yu Miao:** Writing – original draft, Software, Methodology, Formal analysis, Data curation. **Mi-Bo Kim:** Writing – original draft, Visualization, Validation, Investigation. **Suhyeon Baek:** Writing – original draft, Methodology, Formal analysis, Data curation. **Sanggil Lee:** Supervision, Software, Resources, Project administration. **Lei Cao:** Writing – review & editing, Visualization, Supervision, Software, Project administration, Methodology, Investigation, Funding acquisition, Formal analysis, Conceptualization.

## Funding sources

This work was supported by the 10.13039/501100002644Pukyong National University Research Fund in 2025 (202510520001).

## Declaration of competing interest

The authors declare that they have no known competing financial interests or personal relationships that could have appeared to influence the work reported in this paper.

## Data Availability

Data will be made available on request.

## References

[bb0005] Arias R., Lee T.-C., Logendra L., Janes H. (2000). Correlation of lycopene measured by HPLC with the l*, a*, b* color readings of a hydroponic tomato and the relationship of maturity with color and lycopene content. Journal of Agricultural and Food Chemistry.

[bb0010] Baek S.H., Lee J.W., Ho T.C., Park Y., Ata S.M., Yun H.J., Lee S.G. (2025). A comparative study of extraction methods for recovery of bioactive components from brown algae sargassum serratifolium. Food Science and Biotechnology.

[bb0015] Bianchi A.R., Vitale E., Guerretti V., Palumbo G., De Clemente I.M., Vitale L., De Maio A. (2023). Antioxidant characterization of six tomato cultivars and derived products destined for human consumption. Antioxidants (Basel).

[bb0020] Blando F., Berland H., Maiorano G., Durante M., Mazzucato A., Picarella M.E., Andersen O.M. (2019). Nutraceutical characterization of anthocyanin-rich fruits produced by “sun black” tomato line. Frontiers in Nutrition.

[bb0025] Cheng G., Chang P., Shen Y., Wu L., El-Sappah A.H., Zhang F., Liang Y. (2020). Comparing the flavor characteristics of 71 tomato (solanum lycopersicum) accessions in Central Shaanxi. Frontiers in Plant Science.

[bb0030] Cheung A.Y., McNellis T., Piekos B. (1993). Maintenance of chloroplast components during chromoplast differentiation in the tomato mutant green flesh. Plant Physiology.

[bb0035] Del Valle M., Cámara M., Torija M.E. (2006). Chemical characterization of tomato pomace. Journal of the Science of Food and Agriculture.

[bb0040] Ilahy R., Hdider C., Lenucci M.S., Tlili I., Dalessandro G. (2011). Phytochemical composition and antioxidant activity of high-lycopene tomato (solanum lycopersicum l.) cultivars grown in southern Italy. Scientia Horticulturae.

[bb0045] Islam M.T., Lee B.-R., Lee H., Jung W.-J., Bae D.-W., Kim T.-H. (2019). P-Coumaric acid induces jasmonic acid-mediated phenolic accumulation and resistance to black rot disease in brassica napus. Physiological and Molecular Plant Pathology.

[bb0050] Jones P., Vogt T. (2001). Glycosyltransferases in secondary plant metabolism: Tranquilizers and stimulant controllers. Planta.

[bb0055] Joung M., Kim Y.J., Shin Y. (2025). Assessment of lycopene, polyphenols, antioxidant compounds, and activities in colored cherry tomato cultivars harvested in Korea. Food Science and Biotechnology.

[bb0060] Kim D.S., Na H., Kwack Y., Chun C. (2014). Secondary metabolite profiling in various parts of tomato plants. Horticultural Science & Technology.

[bb0065] Kim G. (2018). The Farmers Newspaper.

[bb0070] Kim H.-K., Chun J.-H., Kim S.-J. (2015). Method development and analysis of carotenoid compositions in various tomatoes. Korean Journal of Environmental Agriculture.

[bb0075] Koivikko R., Loponen J., Honkanen T., Jormalainen V. (2005). Contents of soluble, cell-wall-bound and exuded phlorotannins in the brown alga fucus vesiculosus, with implications on their ecological functions. Journal of Chemical Ecology.

[bb0080] Kurina A., Solovieva A., Khrapalova I., Artemyeva A. (2021). Biochemical composition of tomato fruits of various colors. Vavilov journal of genetics and breeding.

[bb0085] Kytidou K., Artola M., Overkleeft H.S., Aerts J.M. (2020). Plant glycosides and glycosidases: A treasure-trove for therapeutics. Frontiers in Plant Science.

[bb0090] Le Roy J., Huss B., Creach A., Hawkins S., Neutelings G. (2016). Glycosylation is a major regulator of phenylpropanoid availability and biological activity in plants. Frontiers in Plant Science.

[bb0095] Li N., Wu X., Zhuang W., Xia L., Chen Y., Wu C., Yi M. (2021). Tomato and lycopene and multiple health outcomes: Umbrella review. Food Chemistry.

[bb0100] Marchiosi R., dos Santos W.D., Constantin R.P., de Lima R.B., Soares A.R., Finger-Teixeira A., Abrahão J. (2020). Biosynthesis and metabolic actions of simple phenolic acids in plants. Phytochemistry Reviews.

[bb0105] Minaker S.A., Mason R.H., Chow D.R. (2021). Optimizing color performance of the ngenuity 3-dimensional visualization system. Ophthalmology Science.

[bb0110] Moco S., Capanoglu E., Tikunov Y., Bino R.J., Boyacioglu D., Hall R.D., De Vos R.C. (2007). Tissue specialization at the metabolite level is perceived during the development of tomato fruit. Journal of Experimental Botany.

[bb0115] Otify A.M., Ibrahim R.M., Abib B., Laub A., Wessjohann L.A., Jiang Y., Farag M.A. (2023). Unveiling metabolome heterogeneity and new chemicals in 7 tomato varieties via multiplex approach of UHPLC-MS/MS, GC–MS, and UV–vis in relation to antioxidant effects as analyzed using molecular networking and chemometrics. Food Chemistry.

[bb0120] Pesaresi P., Mizzotti C., Colombo M., Masiero S. (2014). Genetic regulation and structural changes during tomato fruit development and ripening. Frontiers in Plant Science.

[bb0125] Qin H., Wang Z. (2021). Biogeochemistry of dominant plants and soils in Shewushan gold lateritic deposit, China. Plants.

[bb0130] Schijlen E., Ric de Vos C., Jonker H., Van Den Broeck H., Molthoff J., Van Tunen A., Bovy A. (2006). Pathway engineering for healthy phytochemicals leading to the production of novel flavonoids in tomato fruit. Plant Biotechnology Journal.

[bb0135] Slimestad R., Fossen T., Verheul M.J. (2008). The flavonoids of tomatoes. Journal of Agricultural and Food Chemistry.

[bb0140] Spencer J.P., Kuhnle G.G., Hajirezaei M., Mock H.-P., Sonnewald U., Rice-Evans C. (2005). The genotypic variation of the antioxidant potential of different tomato varieties. Free Radical Research.

[bb0145] Suleria H.A., Barrow C.J., Dunshea F.R. (2020). Screening and characterization of phenolic compounds and their antioxidant capacity in different fruit peels. Foods.

[bb0150] Tudor-Radu M., Vîjan L.E., Tudor-Radu C.M., TiȚA I., Sima R., Mitrea R. (2016). Assessment of ascorbic acid, polyphenols, flavonoids, anthocyanins and carotenoids content in tomato fruits. Notulae botanicae horti agrobotanici Cluj-Napoca.

[bb0155] Wasternack C., Song S. (2017). Jasmonates: Biosynthesis, metabolism, and signaling by proteins activating and repressing transcription. Journal of Experimental Botany.

[bb0160] Yamanaka T., Vincken J.-P., de Waard P., Sanders M., Takada N., Gruppen H. (2008). Isolation, characterization, and surfactant properties of the major triterpenoid glycosides from unripe tomato fruits. Journal of Agricultural and Food Chemistry.

[bb0165] Yilmaz E. (2001). The chemistry of fresh tomato flavor. Turkish Journal of Agriculture and Forestry.

[bb0170] Zhang Y., Fernie A.R. (2023). The role of TCA cycle enzymes in plants. Advanced Biology.

[bb0175] Zhang Y., Zhao G., Li Y., Zhang J., Shi M., Muhammad T., Liang Y. (2017). Transcriptome profiling of tomato uncovers an involvement of cytochrome p450s and peroxidases in stigma color formation. Frontiers in Plant Science.

[bb0180] Zheng H., Torres-Montilla S., Huang X., Rodriguez-Concepcion M., Lu S. (2025). STAY-GREEN overexpression in dark-incubated leaves promotes the formation of transitional chromoplast-like plastids. Plant Physiology.

[bb0185] Zhong P., Wei X., Li X., Wei X., Wu S., Huang W., Lei H. (2022). Untargeted metabolomics by liquid chromatography-mass spectrometry for food authentication: A review. Comprehensive Reviews in Food Science and Food Safety.

[bb0190] Zou J., Li H., Wang Z., Ye M. (2023). Functional characterization of two efficient glycosyltransferases catalysing the formation of rutin from sophora japonica l. Organic & Biomolecular Chemistry.

