# Supplementary material for: Tissue-specific metabolomics and pigment profiling of Black Change and red Chal tomatoes
Source: Food Chem X. 2026 Apr 27;36:103928. doi: 10.1016/j.fochx.2026.103928 (PMC13153461; doi:10.1016/j.fochx.2026.103928)
Supplement: Supplementary file 1 — Supplementary material [file mmc1.docx]

**Tissue-specific metabolomics and pigment profiling of Black Change and red Chal tomatoes**

**List of supplementary materials**

**Table S1.** Tentatively identified metabolites in peel, flesh, and seed tissues of RT and BCT varieties. Samples were extracted with 70% methanol and analyzed by UHPLC-ESI-qTOF-MS/MS in negative ion mode.

**Fig. S1.** Total ion chromatograms of 70% methanol extracts from peel, flesh, and seed tissues of RT and BCT acquired by UHPLC-ESI-MS in negative ion mode.

**Fig. S2.** PCA score plot of metabolites in 70% methanol extracts of peel, flesh, and seed tissues from RT and BCT varieties.

**Fig. S3**. UPLC-MS MRM chromatograms of naringenin chalcone in (A) RT peel and (B) BCT peel.

**Fig. S4.** Lycopene chromatograms of peel, flesh, and seed tissues from RT and BCT analyzed by HPLC-DAD.

**Table S1.** Tentatively identified metabolites in peel, flesh, and seed tissues of RT and BCT varieties. Samples were extracted with 70% methanol and analyzed by UHPLC-ESI-qTOF-MS/MS in negative ion mode.

|  | **Compound** | **m/z** | **Adducts** | **RT (min)** | **Formula** | **MS/MS** | **Class** | |  |
| --- | --- | --- | --- | --- | --- | --- | --- | --- | --- |
| 1 | O-Acetyl-L-serine | 146.0459 | M-H | 0.82 | C_5_H_9_NO_4_ | 146,132,128 | | Amino acids and derivatives | |
| 2 | L-Mannaric acid | 209.0305 | M-H | 0.82 | C_6_H_10_O_8_ | 191,133,87 | Sugar acids | |  |
| 3 | Citric acid | 191.0201 | M-H | 0.87 | C_6_H_8_O_7_ | 191,173 | Organic acids | |  |
| 4 | Aconitic acid | 173.0093 | M-H | 0.87 | C_6_H_6_O_6_ | 173,129,111 | Organic acids | |  |
| 5 | 3-Hydroxy-2-oxo-2H-pyran-6-carboxylic acid | 154.9986 | M-H | 0.87 | C_6_H_4_O_5_ | 129,111 | Organic acids | |  |
| 6 | Citraconic acid | 129.0191 | M-H | 0.87 | C_5_H_6_O_4_ | 129,111,87 | Organic acids | |  |
| 7 | 4-O-α-D-Galactopyranuronosyl-α-D-galactopyranuronat | 367.0524 | M-H | 0.87 | C_14_H_16_N_4_O_4_S_2_ | 367,191 | Organic acids | |  |
| 8 | 7-(5-phospho-α-D-ribosyl)adenine | 346.056 | M-H | 0.9 | C_20_H_13_NO_3_S | 346,191 | Nucleotides | |  |
| 9 | Linolenic Acid | 277.0316 | M-H | 0.9 | C_18_H_30_O_2_ | 277,191 | Fatty acids | |  |
| 10 | 4-(β-L-Xylofuranosyl)-4,6-dihydro-5H-[1,2,3]triazolo[4,5-b]pyridin-5-on | 267.0725 | M-H | 0.9 | C_10_H_12_N_4_O_5_ | 223,191 | Nucleoside analogues | |  |
| 11 | Lipoic acid | 205.0352 | M-H | 0.9 | C_8_H_14_O_2_S_2_ | 205,191,173 | Organic acids | |  |
| 12 | L-Fucono-1,5-lactone | 161.048 | M-H | 0.9 | C_6_H_10_O_5_ | 133,111 | Carbohydrate derivatives | |  |
| 13 | Dihydrogénodiphosphate de [(2R,3S,4R,5R)-5-(2,4-dioxo-3,4-dihydro-1(2H)-pyrimidinyl)-3,4-dihydroxytétrahydro-2-furanyl]méthyle et de (2R,3R,4S,5R,6R)-3,4,5-trihydroxy-6-(hydroxyméthyl)tétrahydro-2H-py ran-2-yle | 565.0483 | M-H | 0.9 | C_15_H_24_N_2_O_17_P_2_ | 565,323 | Nucleotides | |  |
| 14 | Trigalacturonic acid | 545.1011 | M-H | 0.92 | C_18_H_26_O_19_ | 545,317 | Polysaccharide derivatives | |  |
| 15 | Digalacturonic acid | 369.0676 | M-H | 0.92 | C_12_H_18_O_13_ | 369,278,223 | Polysaccharide derivatives | |  |
| 16 | 2-O-Carboxy-D-glucose | 223.0461 | M-H | 0.92 | C_7_H_12_O_8_ | 223,191 | Carbohydrate derivatives | |  |
| 17 | (2R)-2,3-Dihydroxypropyl 6-deoxy-6-sulfo-α-D-glucopyranoside | 317.055 | M-H | 0.95 | C_11_H_15_N_2_O_7_P | 317,225 | Glycosides | |  |
| 18 | 6-O-β-L-Talopyranosyl-β-D-sorbofuranos | 341.1091 | M-H | 1.1 | C_12_H_22_O_11_ | 341,133 | Carbohydrate derivatives | |  |
| 19 | 7-(5-phospho-α-D-ribosyl)adenine | 346.0561 | M-H | 1.5 | C_20_H_13_NO_3_S | 346,134 | Nucleotides | |  |
| 20 | Isocitric acid | 191.0201 | M-H | 1.81 | C_6_H_8_O_7_ | 110,173,191 | Organic acids | |  |
| 21 | 2-Furoic acid | 111.0082 | M-H | 1.81 | C_5_H_4_O_3_ | 111,87 | Organic acids | |  |
| 22 | 5-Oxo-tetrahydro-furan-2-carboxylic acid | 129.0191 | M-H | 1.81 | C_5_H_6_O_4_ | 128,111,87 | Organic acids | |  |
| 23 | 1H-1,2,3-Triazole-1-carboxylate | 111.0082 | M-H | 1.81 | C_3_H_2_N_3_O_2¯_ | 111,87 | Heterocycles | |  |
| 24 | Guanosine | 282.0846 | M-H | 3.39 | C_10_H_13_N_5_O_5_ | 282,150,108 | Nucleosides | |  |
| 25 | (1S,4aS,5R,7S,7aS)-7-Acetoxy-1-(β-D-glucopyranosyloxy)-5-hydroxy-7-methyl-1,4a,5,6,7,7a-hexahydrocyclopenta[c]pyran-4-carboxylic acid | 433.1357 | M-H | 3.47 | C_23_H_22_N_4_O_3_S | 433,271,161 | Glycosides | |  |
| 26 | N-[(2-Amino-4-oxo-1,4-dihydro-7-pteridinyl)carbonyl]-L-seryl-L-tryptophan | 479.1419 | M-H | 3.47 | C_21_H_20_N_8_O_6_ | 479,433 | Peptides | |  |
| 27 | 4-(2-Aminoethyl)benzoic acid | 164.0719 | M-H | 3.82 | C_9_H_11_NO_2_ | 164,147 | Amino acids and derivatives | |  |
| 28 | 2,3,8,9-Tetramethoxy-5-methylbenzo[c]phenanthridin-6(5H)-on | 378.1342 | M-H | 3.99 | C_14_H_25_N_3_O_7_S | 163 | Alkaloids | |  |
| 29 | O-α-D-glucosyl-trans-zeatin | 380.1563 | M-H | 4.23 | C_20_H_23_N_5_OS | 380,191 | Glycosides | |  |
| 30 | Caffeic acid | 179.0350 | M-H | 4.47 | C_9_H_8_O_4_ | 179,135 | Phenolics and polyphenols | |  |
| 31 | 1-Caffeoyl-β-D-glucose | 341.0884 | M-H | 4.47 | C_15_H_18_O_9_ | 341,179,135 | Phenolics and polyphenols | |  |
| 32 | 4-{[(2R,3aS)-3,3a-Dihydro-2H-imidazo[4,5-b]pyridin-2-ylsulfanyl]methyl}-6-ethoxy-2H-chromen-2-on | 354.0917 | M-H | 4.53 | C_18_H_17_N_3_O_3_S | 163,119 | Heterocycles | |  |
| 33 | p-Coumaroylglucose | 325.0931 | M-H | 4.53 | C_15_H_18_O_8_ | 163,119 | Phenolics and polyphenols | |  |
| 34 | Benzoylacetic acid | 163.0401 | M-H | 4.53 | C_9_H_8_O_3_ | 163,119 | Organic Acids | |  |
| 35 | Chlorogenic Acid Hydrate | 371.0986 | M-H | 4.53 | C_16_H_20_O_10_ | 163,119 | Phenolics and polyphenols | |  |
| 36 | D-(+)-tryptophan | 203.0827 | M-H | 4.71 | C_11_H_12_N_2_O_2_ | 203,142,116 | Amino acids and derivatives | |  |
| 37 | 2-(4-{[4-O-(6-Deoxy-α-L-mannopyranosyl)-β-D-xylopyranosyl]oxy}-3,5-dihydroxyphenyl)-5,7-dihydroxy-4-oxo-4H-chromen-3-yl 6-deoxy-2-O-β-D-glucopyranosyl-α-L-mannopyranoside | 903.2427 | M-H | 4.84 | C_56_H_40_O_12_ | 903,741 | Phenolics and polyphenols | |  |
| 38 | 2-Butyryl-3,5-dihydroxyphenyl β-D-glucopyranoside | 357.1180 | M-H | 4.84 | C_16_H_22_O_9_ | 193,179 | Phenolics and polyphenols | |  |
| 39 | (4-Acetylphenoxy)acetic acid | 193.0508 | M-H | 4.88 | C_10_H_10_O_4_ | 193,179,134 | Organic acids | |  |
| 40 | Ferulic acid glucoside | 355.1035 | M-H | 4.88 | C_16_H_20_O_9_ | 193,149,134 | Phenolics and polyphenols | |  |
| 41 | 5-[8-Hydroxy-1,5-dimethyl-3-[3,4,5-trihydroxy-6-(hydroxymethyl)oxan-2-yl]oxy-6-oxabicyclo[3.2.1]octan-8-yl]-3-methylpenta-2,4-dienoic acid | 443.1926 | M-H | 4.95 | C_21_H_32_O_10_ | 443,387 | Polysaccharide derivatives | |  |
| 42 | 4-Acetyl-3-hydroxy-5-methoxyphenyl β-D-glucopyranoside | 327.1030 | M-H | 4.95 | C_15_H_20_O_8_ | 181,101 | Phenolics and polyphenols | |  |
| 43 | N-(4-Acetylphenyl)-2-{[(E)-6,7-dihydro-2,1,3-benzoxadiazol-4(5H)-ylidenamino]oxy}acetamid | 327.1087 | M-H | 5.01 | C_15_H_20_O_8_ | 247,165 | Amides | |  |
| 44 | N-[(2-Hydroxyethyl)(methyl)carbamoyl]-4-(5-methoxy-2-methyl-3-oxo-2,3-dihydro-4-pyridazinyl)-L-phenylalanin | 403.1613 | M-H | 5.01 | C_19_H_24_N_4_O_6_ | 403,247 | Amides | |  |
| 45 | (2S)-2-(1,4-Diazepan-1-yl)-2-(4-hydroxyphenyl)propanoic acid | 263.1403 | M-H | 5.03 | C_14_H_20_N_2_O_3_ | 247,165 | Phenolics and polyphenols | |  |
| 46 | Caffeic acid 3-glucoside | 341.0880 | M-H | 5.08 | C_15_H_18_O_9_ | 341,179,135 | Phenolics and polyphenols | |  |
| 47 | Kaempferol 3-sophorotrioside | 771.2004 | M-H | 5.10 | C_33_H_40_O_21_ | 771,725,609 | Phenolics and polyphenols | |  |
| 48 | D-(−)-Quinic acid | 191.0563 | M-H | 5.15 | C_7_H_12_O_6_ | 191,173,127 | Organic acids | |  |
| 49 | 3-{[(2Z)-3-(3,4-Dioxo-1,5-cyclohexadien-1-yl)-2-propenoyl]oxy}-1,4,5-trihydroxycyclohexanecarboxylic acid | 351.0727 | M-H | 5.15 | C_9_H_16_N_6_O_7_S | 351,191 | Organic acids | |  |
| 50 | Chlorogenic acid | 353.0880 | M-H | 5.15 | C_16_H_18_O_9_ | 353,191 | Phenolics and polyphenols | |  |
| 51 | 6-phospho-β-D-glucosyl-(1->4)-D-glucose | 421.0758 | M-H | 5.15 | C_12_H_23_O_14_P | 375,191 | Phosphorylated disaccharides | |  |
| 52 | 3-[(6-Deoxy-α-L-mannopyranosyl)oxy]-5-hydroxy-2-(4-methoxyphenyl)-4-oxo-4H-chromen-7-yl 6-O-(3-carboxypropanoyl)-β-D-glucopyranoside | 707.1841 | M-H | 5.15 | C_32_H_36_O_18_ | 707,353,191 | Phenolics and polyphenols | |  |
| 53 | 1,2,3,4-Tetra-O-acetyl-1-C-2-propin-1-yl-β-D-glucopyranose | 385.1147 | M-H | 5.18 | C_28_H_19_P | 353,181 | Acetylated carbohydrates | |  |
| 54 | Methyl (1S,4aS,5R,7aS)-5-ethoxy-1-(β-D-glucopyranosyloxy)-7-(hydroxymethyl)-1,4a,5,7a-tetrahydrocyclopenta[c]pyran-4-carboxylate | 431.1563 | M-H | 5.18 | C_19_H_28_O_11_ | 431,353,191 | Glycosides | |  |
| 55 | Scroside D | 477.1625 | M-H | 5.18 | C_38_H_22_ | 431,353,191 | Glycosides | |  |
| 56 | 4-Acetyl-3-hydroxy-5-methoxyphenyl β-D-glucopyranoside | 343.1035 | M-H | 5.19 | C_15_H_20_O_9_ | 343,181 | Phenolics and polyphenols | |  |
| 57 | 3-(4-Hydroxy-3-methoxyphenyl)-3-oxopropyl β-D-glucopyranoside | 357.1193 | M-H | 5.34 | C_16_H_22_O_9_ | 325,163 | Glucosides | |  |
| 58 | 3-CAFFEOYLQUINIC ACID | 353.0879 | M-H | 5.34 | C_16_H_18_O_9_ | 353,325,163 | Phenolics and polyphenols | |  |
| 59 | p-Coumaric acid glucoside | 325.0932 | M-H | 5.34 | C_15_H_18_O_8_ | 325,163,119 | Phenolics and polyphenols | |  |
| 60 | p-Coumaric acid | 163.0402 | M-H | 5.34 | C_9_H_8_O_3_ | 163,119 | Phenolics and polyphenols | |  |
| 61 | Ethyl 3-(β-D-glucopyranosyloxy)butanoate | 293.1244 | M-H | 5.41 | C_12_H_22_O_8_ | 293,131 | Glucosides | |  |
| 62 | N-{4-[(3S)-3-Ethyl-2,6-dioxo-3-piperidinyl]phenyl}-5-[(3aS,4S,6aR)-2-oxohexahydro-1H-thieno[3,4-d]imidazol-4-yl]pentanamid | 457.1931 | M-H | 5.51 | C_31_H_26_N_2_O_2_ | 457,387 | Amides | |  |
| 63 | Tuberonic acid glucoside | 387.1661 | M-H | 5.54 | C_18_H_28_O_9_ | 387,300 | Saponins | |  |
| 64 | (1R,2S)-2,3-Dihydroxy-1-(7-methoxy-2-oxo-2H-chromen-6-yl)-3-methylbutyl β-D-glucopyranoside | 455.1543 | M-H | 5.54 | C_20_H_22_N_7_O_6_^+^ | 447,387 | Glucosides | |  |
| 65 | 2-Hydroxy-7-methyl-3-oxo-1,4,6-cycloheptatriene-1-carboxylic acid | 179.0353 | M-H | 5.59 | C_9_H_8_O_4_ | 179,145,135 | Organic acids | |  |
| 66 | Hydroxy-octanedioic acid derivative | 351.1298 | M-H | 5.61 | C_19_H_20_N_4_OS | 351,300,241 | Fatty acids | |  |
| 67 | 4-(Hydroxymethyl)phenyl-3-O-β-D-glucopyranosyl-β-D-glucopyranosid | 447.1518 | M-H | 5.64 | C_19_H_28_O_12_ | 401,175 | Phenolics and polyphenols | |  |
| 68 | 2-Oxo-2-phenylethyl-2-hydroxy-4-chinolincarboxylat | 306.0770 | M-H | 5.71 | C_18_H_13_NO_4_ | 175,113 | Aromatic heterocycles | |  |
| 69 | 5-Caffeoylquinic acid | 353.0880 | M-H | 5.79 | C_16_H_18_O_9_ | 353,191 | Phenolics and polyphenols | |  |
| 70 | Tuberonic acid glucoside | 387.1662 | M-H | 5.84 | C_19_H_24_N_4_O_5_ | 387,191 | Saponins | |  |
| 71 | Dihydroxy-megastigmadien-9-one hexoside (Citroside A) | 385.1927 | M-H | 5.96 | C_19_H_30_O_8_ | 421,365 | Terpenoids | |  |
| 72 | N-[(S)-{(2S)-1-[(2S)-2-Amino-3-carboxypropanoyl]-2-pyrrolidinyl}(carboxy)methyl]-L-alanyl-L-prolin | 427.1826 | M-H | 6.02 | C_22_H_28_N_4_O_3_S | 427,381 | Peptides | |  |
| 73 | Quercetin 3-(2G-xylosylrutinoside) | 741.1893 | M-H | 6.34 | C_32_H_38_O_20_ | 300,740 | Phenolics and polyphenols | |  |
| 74 | (4S)-4-(4-Butoxyphenyl)-1-(6-methoxy-1,3-benzothiazol-2-yl)-3-methyl-1,4,5,7-tetrahydro-6H-pyrazolo[3,4-b]pyridin-6-one | 461.1672 | M-H | 6.40 | C_25_H_26_N_4_O_3_S | 461,284 | Heterocycles | |  |
| 75 | Camelliaside B | 725.1946 | M-H | 6.74 | C_32_H_38_O_19_ | 725,609 | Glycosides | |  |
| 76 | Rutin | 609.1465 | M-H | 6.74 | C_27_H_30_O_16_ | 609,300,151 | Phenolics and polyphenols | |  |
| 77 | Lycoperoside F/G | 1268.595 | M-H | 6.90 | C_58_H_95_NO_29_ | 1268 | Saponins | |  |
| 78 | (3S,3aS,6E,9S,11aS)-6-(Hydroxymethyl)-3,10-dimethyl-2-oxo-2,3,3a,4,5,8,9,11a-octahydrocyclodeca[b]furan-9-yl β-D-glucopyranoside | 427.1975 | M-H | 6.95 | C_22_H_28_N_4_O_5_ | 427,205,153 | Glycosides | |  |
| 79 | Phloretin 3',5'-Di-C-glucoside | 597.1830 | M-H | 7.03 | C_27_H_34_O_15_ | 597,387,357 | Phenolics and polyphenols | |  |
| 80 | DL-N-Acetyltryptophan（Pimpinellin） | 245.0935 | M-H | 7.23 | C_13_H_14_N_2_O_3_ | 245,203 | Amino acids and derivatives | |  |
| 81 | Lycoperoside F/G | 1268.594 | M-H | 7.25 | C_58_H_95_NO_29_ | 1268 | Saponins | |  |
| 82 | Lycoperoside F derivative | 1291.598 | M-H | 7.25 | C_58_H_96_NO_30_ | 1268,1136 | Saponins | |  |
| 83 | Kaempferol 3-O-robinobioside | 593.1517 | M-H | 7.25 | C_27_H_30_O_15_ | 593,285,179 | Phenolics and polyphenols | |  |
| 84 | 3,4-Dicaffeoylquinic acid | 515.1205 | M-H | 7.38 | C_25_H_24_O_12_ | 353,179 | Phenolics and polyphenols | |  |
| 85 | L-Alanyl-L-threonyl-L-prolyl-L-phenylalaninamid | 432.2246 | M-H | 7.43 | C_21_H_31_N_5_O_5_ | 432,387 | Peptides | |  |
| 86 | 3-Cyclopentyl-N-[(1S,2R)-2-(N-hydroxycarbamimidoyl)cyclopentyl]propanamid | 266.1878 | M-H | 7.56 | C_14_H_25_N_3_O_2_ | 266,140 | Amides | |  |
| 87 | Quercetin 3-(sinapoyl-pentosyl-rhamnosyl)-hexoside | 947.2484 | M-H | 7.60 | C_43_H_48_O_24_ | 947,741 | Phenolics and polyphenols | |  |
| 88 | Anhydrolutein-I | 549.1987 | M-H | 7.62 | C_27_H_34_O_12_ | 549,387 | Saponins | |  |
| 89 | (1S,2S,2aR,5S,5aS,5bR,6aS,6bS,7R,8aS,9aS,10aS,11R,11aR,11bS,12S,13R,13bS)-2,2a,5,6,7-Pentahydroxy-1,5,5a,11a,13a-pentamethyl-4,8-dioxodocosahydro-1H-oxireno[6′,7′]naphtho[1′,2′:7,8]fluoreno[2,1-b]fura n-11,12,13-triyl triacetate | 693.2788 | M-H | 7.73 | C_34_H_46_O_15_ | 693,675 | Alkenoic acid derivatives | |  |
| 90 | Naringenin 4′-O-glucoside | 433.1148 | M-H | 7.78 | C_21_H_22_O_10_ | 277,151 | Phenolics and polyphenols | |  |
| 91 | Kaempferol 3-O-[6-(4-coumaroyl)-β-D-glucosyl-(1->2)-β-D-glucosyl-(1->2)-β-D-glucoside] | 917.2373 | M-H | 7.80 | C_42_H_46_O_23_ | 917,887,741 | Phenolics and polyphenols | |  |
| 92 | Kaempferol 3-apioside-7-rhamnosyl-(1->6)-(2''-(E)-caffeoylglactoside) | 887.2261 | M-H | 7.80 | C_41_H_44_O_22_ | 887,741,300 | Phenolics and polyphenols | |  |
| 93 | 3,4-Dicaffeoylquinic acid | 515.1200 | M-H | 7.86 | C_25_H_24_O_12_ | 515,353,173 | Phenolics and polyphenols | |  |
| 94 | Caffeic acid 3-sophoroside | 503.178 | M-H | 8.04 | C_24_H_32_N_4_O_4_S_2_ | 448,163 | Phenolics and polyphenols | |  |
| 95 | 1-Acetyl-L-prolyl-L-leucyl-L-histidyl-L-seryl-O-phosphono-L-threoninamid | 673.2725 | M-H | 8.04 | C_42_H_50_O_23_ | 673,617,585 | Peptides | |  |
| 96 | Isohemiphloin | 433.1145 | M-H | 8.49 | C_21_H_22_O_10_ | 271,163 | Phenolics and polyphenols | |  |
| 97 | Kaempferol 3-apioside-7-rhamnosyl-(1->6)-(2''-(E)-caffeoylglactoside) derivative | 921.2682 | M-H | 8.49 | C_42_H_46_O_23_ | 271,300,723,  741,877,000 | Phenolics and polyphenols | |  |
| 98 | 3,4,5-Tricaffeoylquinic acid | 677.2838 | M-H | 8.58 | C_35_H_42_N_4_O_10_ | 677,645,520,  488 | Phenolics and polyphenols | |  |
| 99 | (8-Hydroxy-6-methoxy-4,5-dimethyl-1-oxo-1H-isochromen-3-yl)methyl 4-O-methyl-β-D-glucopyranoside | 425.1458 | M-H | 8.69 | C_20_H_26_O_10_ | 179,135 | Glycosides | |  |
| 100 | Tomatoside A | 1081.5470 | M-H | 8.79 | C_51_H_86_O_24_ | 1081,919,757 | Terpenoids | |  |
| 101 | Moupinamide | 312.1243 | M-H | 8.82 | C_18_H_19_NO_4_ | 312,178,163 | Amides | |  |
| 102 | 1,3,5-Tricaffeoylquinic acid | 677.1522 | M-H | 9.26 | C_34_H_30_O_15_ | 677,515 | Phenolics and polyphenols | |  |
| 103 | β-D-Glucopyranoside, (2E,4R)-4-hydroxy-3,7-dimethyl-2,6-octadien-1-yl 6-O-α-D-glucopyranosyl- | 493.2300 | M-H | 9.34 | C_27_H_34_N_4_O_3_S | 447,223 | Glycosides | |  |
| 104 | Ptychantin J/k | 495.2605 | M-H | 10.02 | C_27_H_36_N_4_O_5_ | 495,453,435 | Glycosides | |  |
| 105 | Apigenin 7-di-O-xyloside | 565.1122 | M-H | 10.17 | C_32_H_22_O_10_ | 565,271 | Phenolics and polyphenols | |  |
| 106 | Naringenin chalcone | 271.0619 | M-H | 10.17 | C_17_H_20_O_3_ | 271,151,119 | Phenolics and polyphenols | |  |
| 107 | Naringenin chalcone noncovalent dimer | 543.1302 | M-H | 10.17 | C_30_H_24_O_10_ | 543,271,151,  119 | Phenolics and polyphenols | |  |
| 108 | Ptychantin J | 495.2605 | M-H | 10.31 | C_28_H_40_N_4_S_2_ | 495,453,435 | Glycosides | |  |
| 109 | (3α,5β)-17,21-Dihydroxy-11,20-dioxopregnan-3-yl β-D-glucopyranosiduronic acid | 539.2508 | M-H | 10.31 | C_27_H_40_O_11_ | 495,453,435 | Glycosides | |  |
| 110 | O-[(1R,2R,5R)-2-Isopropyl-5-methylcyclohexyl]-L-serine* | 242.1759 | M-H | 10.85 | C_13_H_25_NO_3_ | 242,225,181 | Amino acids and derivatives | |  |
| 111 | (−)-pinellic acid* | 329.2335 | M-H | 11.19 | C_18_H_34_O_5_ | 329,211,171 | Organic acids | |  |
| 112 | (3S,12S)-3,12-Dihydroxyhexadecanoic acid | 287.2230 | M-H | 11.51 | C_16_H_32_O_4_ | 287,255 | Fatty acids | |  |
| 113 | (12E,14R)-14-Hydroperoxy-12-octadecenoic acid | 313.2388 | M-H | 12.21 | C_18_H_34_O_4_ | 279,255 | Fatty acids | |  |
| 114 | 7,10,12-trihydroxy-8-octadecenoic acid | 329.2335 | M-H | 12.52 | C_18_H_34_O_5_ | 329,201 | Fatty acids | |  |
| 115 | β-Phocaecholate | 407.2802 | M-H | 12.97 | C_24_H_40_O_5_ | 407,279 | Organic acids | |  |
| 116 | Fusarilactone B | 309.1709 | M-H | 13.79 | C_17_H_26_O_5_ | 309,265,209,  152 | Phenolics and polyphenols | |  |
| 117 | Hydroxy-octadecatrienoic acid | 293.1766 | M-H | 14.62 | C_17_H_26_O_4_ | 293,249,193 | Fatty acids | |  |



 **Figure S1.** Total ion chromatograms of 70% methanol extracts from peel, flesh, and seed tissues of RT and BCT acquired by UHPLC-ESI-MS in negative ion mode.


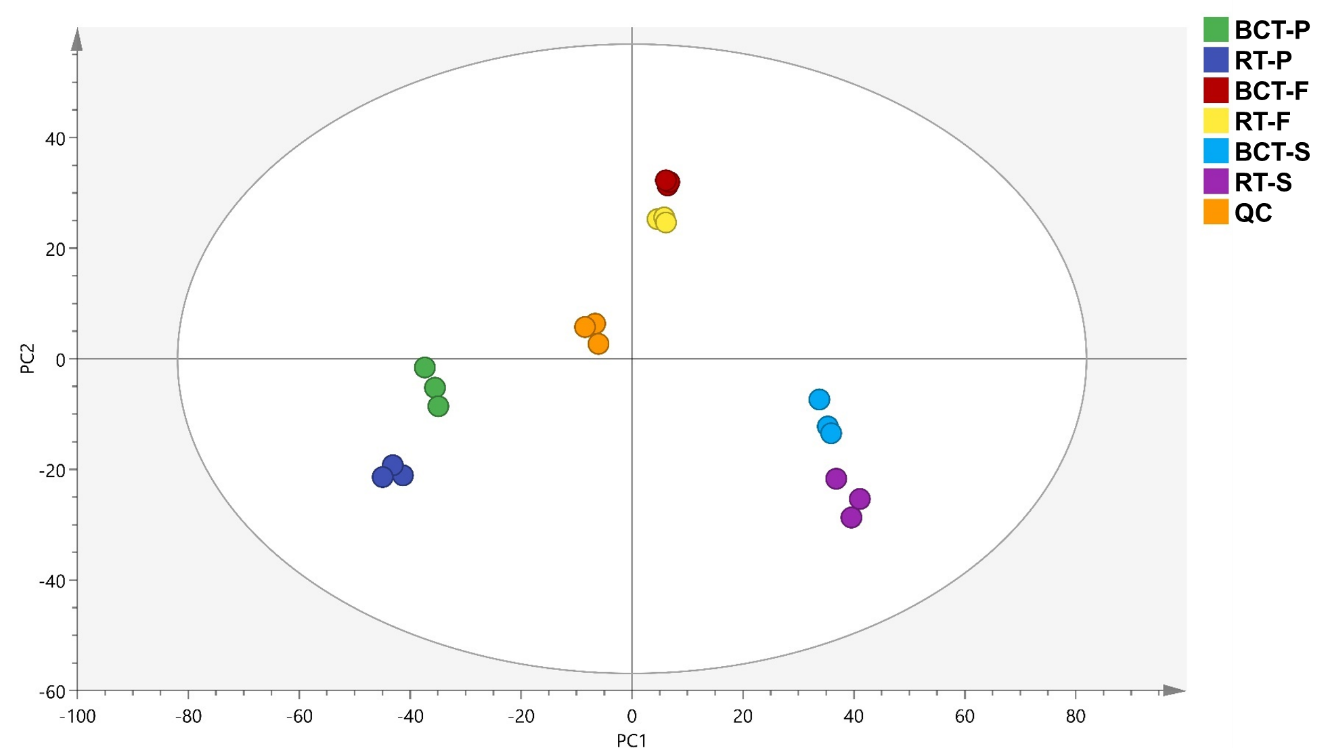


Figure S2. PCA score plot of metabolites in 70% methanol extracts of peel, flesh, and seed tissues from RT and BCT varieties. BCT-P, Black Change tomato peel; RT-P, red tomato peel; BCT-F, Black Change tomato flesh; RT-F, red tomato flesh; BCT-S, Black Change tomato seed; RT-S, red tomato seed.


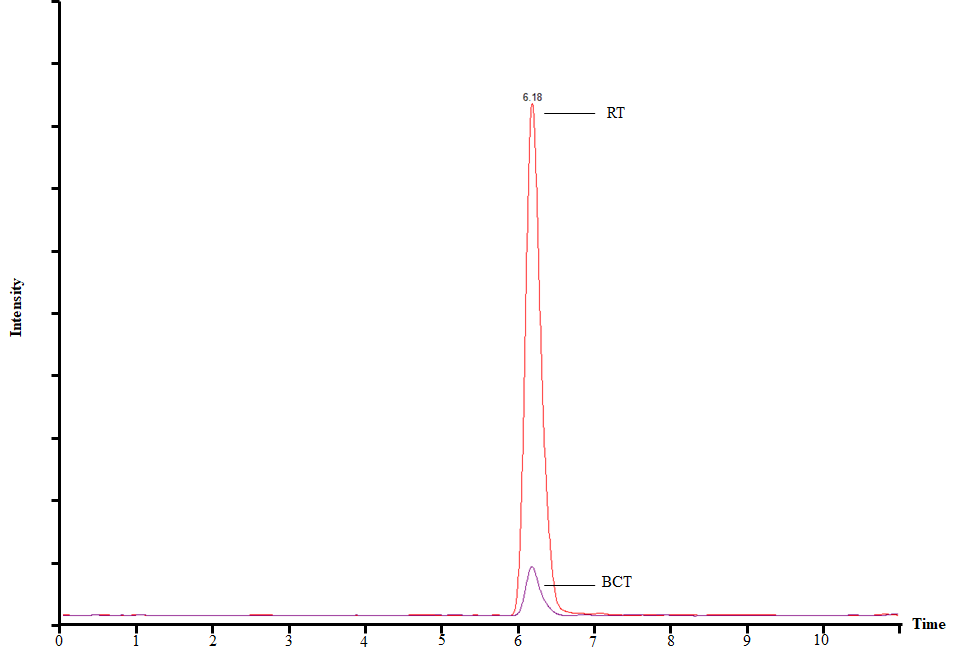


Figure S3. UPLC-MS MRM chromatograms of naringenin chalcone in RT peel and BCT peel.


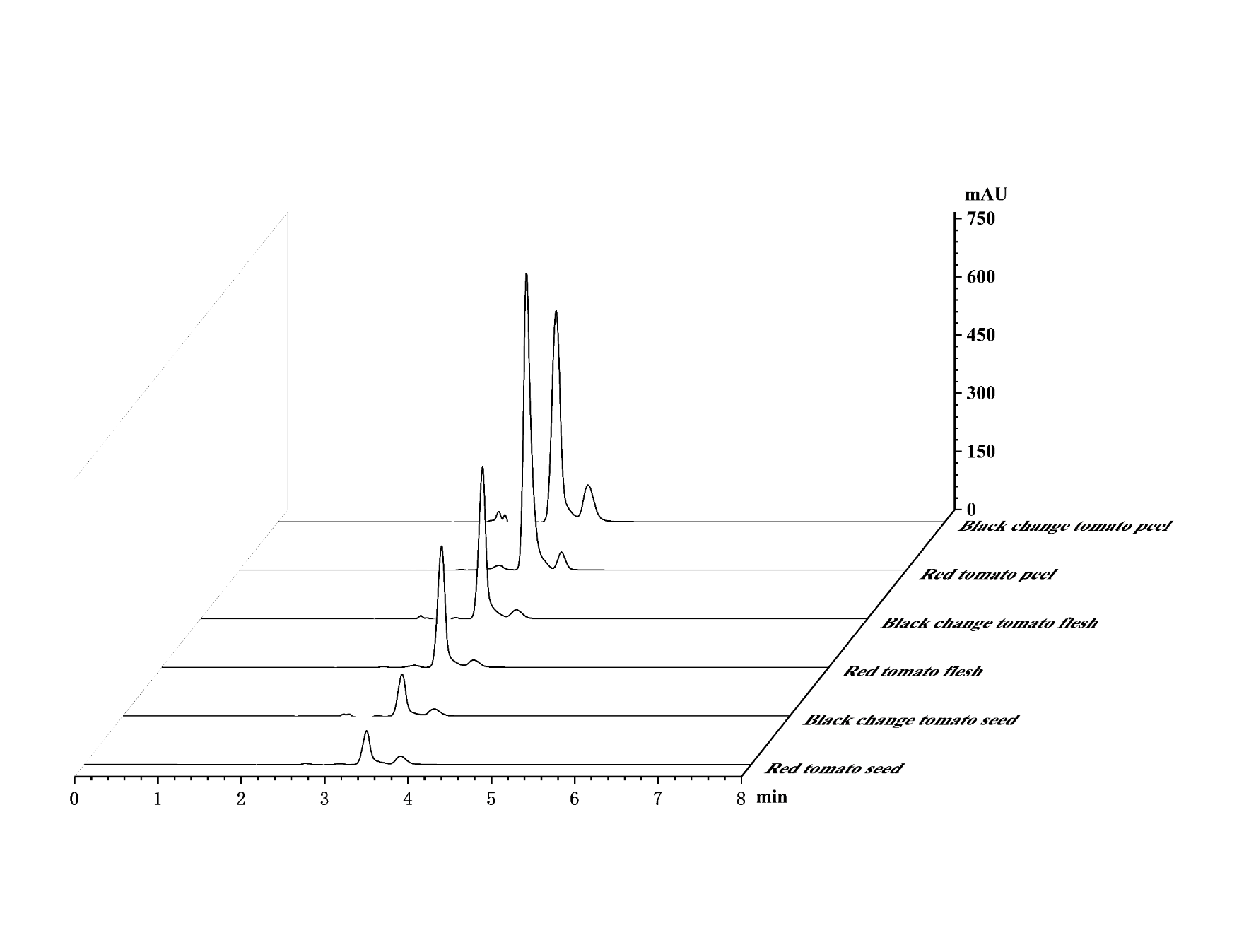


Figure S4. Lycopene chromatograms of peel, flesh, and seed tissues from RT and BCT analyzed by HPLC-DAD.
